# Comparative evaluation of total intravenous anaesthesia versus inhalational anaesthesia on postoperative recovery in elective abdominal surgery

**DOI:** 10.6026/973206300220927

**Published:** 2026-02-28

**Authors:** Aditya Singh Chauhan, Brajesh Kumar, Nancy Ekka, Sourabh Shrivastava

**Affiliations:** 1 Department of Anaesthesiology, Gajra Raja Medical college Gwalior, Madhya Pradesh, India; 2 Consultant Anaesthesiologist, Bansal Hospital, Sagar, Madhya Pradesh, India

**Keywords:** Total intravenous anaesthesia (TIVA), propofol, remifentanil, sevoflurane, postoperative recovery, postoperative nausea and vomiting, emergence agitation, patient satisfaction

## Abstract

The selection of anaesthetic technique markedly influences the quality and speed of postoperative recovery after abdominal surgery, yet no consensus exists on the optimal approach. Therefore, it is of interest to compare total intravenous anaesthesia (TIVA) using propofol-remifentanil with sevoflurane-fentanyl inhalational anaesthesia in 100 adults undergoing elective abdominal surgery. TIVA patients demonstrated significantly faster emergence, with shorter times to eye opening (7.1±2.3 vs 10.2±3.1 min) and extubation (8.8±2.5 vs 12.7±3.4 min; p<0.001). Post-anaesthesia care unit (PACU) stay was reduced in the TIVA group (54.8±13.2 vs 71.6±17.4 min; p<0.001) alongside lower postoperative nausea and vomiting (16% vs 40%) and agitation (8% vs 24%). Patient satisfaction was higher with TIVA (8.8±1.0 vs 7.5±1.5; p<0.001), with postoperative pain and opioid consumption remaining comparable between groups. Overall, propofol-remifentanil TIVA offered faster, smoother recovery and greater comfort without compromising analgesia in elective abdominal surgeries.

## Background:

General anaesthesia can be maintained by either intravenous or inhalational techniques, each offering distinct pharmacokinetic and pharmacodynamic profiles that influence postoperative recovery [1]. Inhalational agents have traditionally dominated clinical practice because of their titratability, rapid onset-offset with modern compounds and familiarity [2]. However, total intravenous anaesthesia (TIVA), particularly propofol-remifentanil combinations delivered via target-controlled infusion (TCI), has gained considerable traction due to favourable emergence characteristics, intrinsic antiemetic effects and absence of operating-theatre pollution [3, 4]. Postoperative nausea and vomiting remains one of the most unpleasant complications after abdominal surgery, with baseline risk often exceeding 50% in the absence of prophylaxis [5]. Volatile anaesthetics are independent risk factors for PONV, whereas propofol possesses dose-dependent antiemetic properties mediated through serotonin receptor modulation and gabaergic mechanisms [6, 7]. Multiple meta-analyses have confirmed that maintenance with propofol reduces PONV odds by 35-45% compared with volatile agents [8, 9]. Emergence quality is another critical consideration. Sevoflurane, despite its low blood-gas solubility, demonstrates context-sensitive decrement times that prolong recovery after infusions exceeding 2-3 hours, whereas propofol's rapid redistribution and hepatic clearance permit swift return of consciousness even after prolonged administration [10]. Clinical trials in ambulatory and day-case surgery consistently report 2-5-minute shorter extubation times and earlier PACU discharge with TIVA [11, 12]. Emergence agitation and delirium, though more widely studied in paediatric populations, also occur in adults and are associated with volatile anaesthetics, particularly after visceral stimulation [13]. Propofol-based techniques appear to produce smoother emergence and lower agitation scores [14]. Despites these theoretical advantages, comparative data specific to abdominal surgery-where peritoneal irritation, opioid requirements and prolonged duration compound recovery challenges-remain limited and sometimes contradictory [15]. Many previous trials used older volatile agents, non-TCI propofol delivery, or heterogeneous surgical populations, reducing applicability to contemporary practice. Therefore, it is of interest to compare propofol-remifentanil TIVA versus sevoflurane-fentanyl inhalational anaesthesia in a homogeneous cohort of adults undergoing elective abdominal surgery with respect to early recovery milestones, PONV, pain, emergence agitation and patient satisfaction.

## Materials and Methods:

This prospective, randomised, parallel-group, assessor-blinded trial was conducted between January 2022 and June 2023. Sample size was calculated to detect a 3-minute difference in extubation time (expected SD 4.5 min, α = 0.05, power 90%), yielding 46 patients per group. Accounting for 10% dropout, 50 patients per group were recruited. Adults aged 18-65 years, ASA physical status I-II, BMI 18-32 kg/m^2^, scheduled for elective open or laparoscopic abdominal procedures expected to last 60-180 minutes were eligible. Exclusion criteria comprised known allergy to study drugs, significant cardiovascular, respiratory, hepatic or renal disease, chronic opioid or antiemetic use, history of severe PONV or motion sickness, pregnancy and anticipated difficult airway. Computer-generated randomisation (1:1 ratio, block size 8) was performed by an independent statistician. Allocation was concealed using sequentially numbered opaque sealed envelopes opened in the operating theatre. Anaesthetists were necessarily unblinded, but postoperative assessors and patients remained blinded to group assignment. All patients received standardised premedication with intravenous ondansetron 4 mg and ranitidine 50 mg 30 minutes pre-induction. Monitoring included ECG, non-invasive blood pressure, pulse oximetry, capnography, neuromuscular monitoring (TOF-Watch) and bispectral index (BIS Vista). Anaesthesia depth was titrated to maintain BIS 40-60.

## TIVA group:

Induction and maintenance used propofol TCI (Marsh pharmacokinetic model, initial plasma target 4-6 µg/ml) and remifentanil TCI (Minto model, effect-site target 4-8 ng/ml). Rocuronium 0.6 mg/kg facilitated tracheal intubation; additional boluses were given as required.

## Inhalation group:

Induction used propofol 2-2.5 mg/kg and fentanyl 2µg/kg. Maintenance was with sevoflurane in oxygen-air mixture (fio_2_ 0.5), adjusted to 0.9-1.3 age-corrected MAC with intermittent fentanyl 0.5-1 µg/kg boluses to maintain haemodynamic stability and BIS targets. Ventilation was volume-controlled (tidal volume 6-8 ml/kg ideal body weight, PEEP 5-8 cmh_2_O). Normothermia and normocapnia were maintained. At skin closure, anaesthetic agents were discontinued simultaneously and neuromuscular blockade was reversed with neostigmine50 µg/kg and glycopyrrolate 10µg/kg when TOF ratio ≥0.9. Primary outcomes were time from anaesthetic discontinuation to spontaneous eye opening on verbal command, tracheal extubation and achievement of modified Aldrete score ≥9. Secondary outcomes included PONV incidence and severity (0-24 h), rescue antiemetic requirements, visual analogue scale (VAS) pain scores at 0, 2, 6 and 24h, 24-hour morphine consumption, emergence agitation (Riker Sedation-Agitation Scale ≥5) and patient satisfaction (0-10 numerical rating scale) at 24h. Data were analysed using SPSS version 26.0 (IBM). Continuous variables are presented as mean ± SD or median (IQR) according to distribution; categorical variables as numbers and percentages. Student's t-test or Mann-Whitney U test and chi-square/Fisher's exact test were used as appropriate. P < 0.05 was considered statistically significant.

## Results:

One hundred patients were randomised and all completed the study. Baseline demographic and surgical characteristics were comparable between groups (Table 1). Intraoperative haemodynamics, total fentanyl/remifentanil consumption (expressed as fentanyl equivalents) and BIS values were similar. Patients in the TIVA group demonstrated significantly faster recovery across all primary endpoints (Table 2). Postoperative pain scores and 24-hour opioid consumption were comparable between groups, while TIVA patients demonstrated significantly lower emergence agitation and higher satisfaction scores (Table 3).

## Discussion:

This randomised controlled trial demonstrates clear superiority of propofol-remifentanil TIVA over sevoflurane-fentanyl inhalational anaesthesia for early postoperative recovery after elective abdominal surgery. The observed 3.1-minute reduction in eye-opening time and 3.9-minute shorter extubation time translate into clinically meaningful earlier return of protective reflexes and reduced airway instrumentation duration. These findings align with the pharmacokinetic advantages of propofol, which exhibits rapid clearance independent of duration of infusion, whereas sevoflurane demonstrates gradual washout from adipose tissue during longer procedures [10, 16]. The 23% shorter PACU stay represents substantial resource saving and improved throughput. The 60% relative reduction in PONV incidence is consistent with extensive literature confirming propofol's antiemetic efficacy [8, 9 and 17]. Avoidance of volatile agents eliminates direct stimulation of the chemoreceptor trigger zone and area postrema, while propofol's GABA-mediated inhibition of serotonergic pathways provides additional protection [7]. Given that abdominal surgery itself confers high emetogenic risk, the ability of TIVA to halve PONV rates despite standardised prophylaxis has major implications for patient experience and cost. Emergence agitation was reduced by two-thirds with TIVA, corroborating reports that volatile anaesthetics disrupt cortical integration more than intravenous agents during the recovery phase [13, 14]. Smoother emergence likely contributed to the significantly higher patient satisfaction scores observed in the TIVA group. Notably, postoperative pain and opioid requirements were equivalent, indicating that ultra-short-acting remifentanil does not precipitate rebound hyperalgesia when used within a multimodal analgesic regimen including local anaesthetic wound infiltration and regular paracetamol/NSAIDs. This contradicts earlier concerns raised in shorter procedures without adequate transition analgesia. Strengths of the study include strict standardisation of surgical type and duration, use of TCI systems ensuring precise drug delivery, BIS-guided anaesthesia eliminating depth differences and blinded outcome assessment. Limitations include inability to blind the anaesthetist, single-centre design and exclusion of higher-risk (ASA III-IV) patients, limiting generalisability to more complex cases.

## Conclusion:

In adults undergoing elective abdominal surgery of moderate duration, propofol-remifentanil TIVA ensures faster emergence, shorter PACU stay and markedly reduced PONV and agitation. It also delivers higher patient satisfaction compared with sevoflurane-fentanyl inhalational anaesthesia, with equivalent postoperative analgesia. These benefits support preferential TIVA use when appropriate expertise and equipment are available.

## Figures and Tables

**Table 1:** Patient demographics and surgical characteristics

**Parameter**	**TIVA (n=50)**	**Inhalational (n=50)**	**P-value**
Age (years)	43.8±12.4	45.1±11.9	0.592
Gender (M/F)	28/22	26/24	0.841
BMI (kg/m^2^)	25.6±3.8	26.1±4.1	0.513
ASA I/II	32/18	29/21	0.683
Surgical procedure (open/laparoscopic)	21/29	23/27	0.841
Duration of surgery (min)	118±34	122±37	0.576
Duration of anaesthesia (min)	142±38	147±41	0.514

**Table 2:** Primary recovery endpoints and PONV outcomes

**Parameter**	**TIVA (n=50)**	**Inhalational (n=50)**	**P-value**
Time to eye opening (min)	7.1±2.3	10.2±3.1	<0.001
Time to extubation (min)	8.8±2.5	12.7±3.4	<0.001
PACU discharge time (min)	54.8±13.2	71.6±17.4	<0.001
PONV incidence 0-24 h, n (%)	8 (16%)	20 (40%)	0.009
Severe PONV requiring >1 rescue antiemetic, n (%)	3 (6%)	11 (22%)	0.022

**Table 3:** Secondary clinical outcomes

**Parameter**	**TIVA (n=50)**	**Inhalational (n=50)**	**P-value**
VAS pain score at PACU arrival	2.3±1.1	2.5±1.2	0.418
VAS at 2 h	3.5±1.3	3.8±1.4	0.281
VAS at 6 h	2.9±1.2	3.2±1.3	0.309
VAS at 24 h	1.7±0.8	1.9±0.9	0.312
24-h morphine consumption (mg)	13.8±6.2	15.4±7.1	0.264
Emergence agitation (Riker ≥5), n (%)	4 (8%)	12 (24%)	0.038
Patient satisfaction score (0-10)	8.8±1.0	7.5±1.5	<0.001
